# Voxel‐based meta‐analysis of gray and white matter volume abnormalities in spinocerebellar ataxia type 2

**DOI:** 10.1002/brb3.1099

**Published:** 2018-08-20

**Authors:** Qing Han, Jing Yang, Hai Xiong, Huifang Shang

**Affiliations:** ^1^ Department of Neurology West China Hospital Sichuan University Chengdu, Sichuan China; ^2^ Department of Geriatrics The Fourth Affiliated Hospital of Sichuan University Chengdu, Sichuan China

**Keywords:** gray matter, spinocerebellar ataxia type 2, volume abnormalities, voxel‐based meta‐analysis, white matter

## Abstract

**Objective:**

To identify the consistent findings from the whole‐brain voxel‐based morphometry (VBM) studies on spinocerebellar ataxia type 2 (SCA2).

**Methods:**

The whole‐brain VBM studies comparing SCA2 patients and healthy controls (HCs) were systematically searched in PubMed, Embase databases from January 2000 to June 2017. The coordinates with significant differences in gray matter (GM) and white matter (WM) between SCA2 patients and HCs were extracted separately from each cluster. A meta‐analysis was performed using anisotropic effect size‐based signed differential mapping (AES‐SDM) software.

**Results:**

A total of five studies with 65 SCA2 patients and 124 HCs were included in the GM meta‐analysis. Four of the five studies with 50 SCA2 patients and 109 HCs were included in the WM meta‐analysis. Significant and consistent GM volume reductions were detected in bilateral cerebellar hemispheres, cerebellar vermis, the right fusiform gyrus, the right parahippocampal gyrus, and the right lingual gyrus. The WM volume reductions were observed in bilateral cerebellar hemispheres, cerebellar vermis, middle cerebellar peduncles, pons, and bilateral cortico‐spinal projections. The findings of the study remained largely unchanged in jackknife sensitivity analysis.

**Conclusions:**

The consistent findings from our meta‐analysis showed that GM volume reductions in SCA2 patients were not limited in cerebellum while significant WM volume reductions widely existed in cerebellum and pyramidal system. The findings provide morphological basis for further studies on SCA2.

## 1 INTRODUCTION

The autosomal dominant cerebellar ataxias (ADCAs), called as spinocerebellar ataxias (SCAs), are a heterogeneous group of chronic and progressive neurodegenerative disorders principally characterized by cerebellar ataxia. Due to the genetic heterogeneity, patients with SCAs can develop impaired vision, dysarthria, pyramidal signs, ophthalmoplegia, extrapyramidal signs, loss of sensory function, dementia, or any combination of these abnormalities (Rossi et al., 2014; van Gaalen, Giunti, & van de Warrenburg, 2011). Among these, spinocerebellar ataxia type 2 (SCA2), one of the most frequent types, is definitely caused by an CAG repeat expansion in the *ATXN2* gene (Pulst et al., 1996). Although the main clinical features of SCA2 are a series of cerebellar signs, including ataxic gait, dysarthria, and dysmetria, which highlight the involvement of cerebellum, some other symptoms such as slow saccades, cognitive impairments, peripheral neuropathy, and depression indicate the abnormalities are beyond the cerebellum (Pulst et al., 1996; Rodríguez‐Labrada et al., 2016; Schmitz‐Hübsch et al., 2011; van Gaalen et al., 2011). The morphological study is essential to detect affected areas of SCA2 while exploring associations between affected areas in the whole brain and clinical manifestations of SCA2 may help us more deeply understand its pathophysiological mechanism, which has remained unknown until now.

Previous neuropathological findings have shown extensive atrophy of cerebellum, brainstem, basal ganglia, and frontal lobes in SCA2 patients (Estrada, Galarraga, Orozco, Nodarse, & Auburger, 1999; Martin et al., 1994), but these postmortem studies may not well reflect abnormalities in vivo or the central nervous system alteration with the disease progression. Voxel‐based morphometry (VBM), a processing technique of magnetic resonance imaging (MRI) that can detect regional morphological changes in the whole brain, has been commonly used to evaluate gray matter (GM) and white matter (WM) volume abnormalities in SCA2 patients (Brenneis, Bosch, Schocke, Wenning, & Poewe, 2003; D'Agata, Caroppo, Boghi et al., 2011; Goel et al., 2011; Hernandez‐Castillo et al., 2015; Jacobi et al., 2013; Mercadillo et al., 2014; Nave, Ginestroni, Tessa, Cosottini et al., 2008; Nave, Ginestroni, Tessa, Salvatore et al., 2008; Olivito et al., 2018). However, the findings of these studies are variable and conflicting. Some studies reported regional cerebral atrophy in frontal, parietal, and temporal lobes (Brenneis et al., 2003; Goel et al., 2011; Hernandez‐Castillo et al., 2015; Mercadillo et al., 2014; Nave, Ginestroni, Tessa, Salvatore et al., 2008), while some studies found brain volume reductions limited in the infratentorial regions such as cerebellum and pons (D'Agata, Caroppo, Boghi et al., 2011; Jacobi et al., 2013; Nave, Ginestroni, Tessa, Cosottini et al., 2008; Olivito et al., 2018). The atrophy of mesencephalon and thalamus was also detected in a few studies (Brenneis et al., 2003; Nave, Ginestroni, Tessa, Salvatore et al., 2008), and significant parahippocampal atrophy was demonstrated in one study (Mercadillo et al., 2014). Although all of these neuroimaging studies reported GM and WM volume reductions in the cerebellum in SCA2, the neurological hallmark for SCAs, the predominant side and regions of cerebellum involved were inconsistent.

Therefore, a meta‐analysis to identify the robust and consistent brain changes in SCA2 patients is essential. The anisotropic effect size‐based signed differential mapping (AES‐SDM) is a quantitative voxel‐based meta‐analytic tool for neuroimaging studies that has been widely used in various neurodegenerative diseases (Pan, Song, & Shang, 2012; Shen et al., 2016; Wang et al., 2015; Yang, Shao, Li, & Shang, 2014). The present meta‐analysis aims to identify the consistent GM and WM changes in SCA2 that had been detected in published individual studies using the AES‐SDM method.

## 2 METHODS

### 2.1 Data source and study selection

Systematic searches were conducted from January 2000 to June 2017 in the PubMed and EMBASE database using the combined keywords (“spinocerebellar ataxia type 2” OR “SCA2” OR “spinocerebellar ataxia 2”) and (“voxel‐based” OR “VBM” OR “morphometry”). An additional search was also conducted in the reference list of relevant articles.

Studies were considered for inclusion if they (a) reported the whole‐brain VBM results of GM volume or WM volume from a comparison between SCA2 patients and HCs; (b) reported the whole‐brain results of changes in a standard stereotactic space with three‐dimensional coordinates (x, y, z); (c) used significance thresholds corrected for multiple comparisons or those uncorrected with spatial extent thresholds; and (d) were peer‐reviewed and published in English. For those studies that met the aforementioned inclusion criteria with overlapping samples, only the study with the largest sample size was included to avoid repetitive data. Studies were excluded if (a) there was no HC group; (b) stereotactic coordinates of the reported changes in the whole brain were not obtained even if we corresponded to authors by email; and (c) the data overlapped with those of another article.

### 2.2 Data extraction

In each study, coordinates and their effect sizes (*t* statistics, *z* scores or *p* values) with significant differences between patients and HCs in GM and WM volume were extracted by two neurologists (namely, Qing Han and Jing Yang) independently according to the AES‐SDM software tutorial. If there were disagreements, the third neurologist (Huifang Shang) would check the data and make a decision finally.

### 2.3 Meta‐analysis

The meta‐analysis of included studies was performed in a standard process using the SDM software package (http://www.sdmproject.com) to compare the differences of GM and WM volume between SCA2 patients and HCs. In brief, we first converted all the other effect size (*z* scores and *p* values) into *t* statistics and then entered the data obtained into the SDM software. The GM volume analysis and WM volume analysis were performed separately and used different masks. Standard MNI maps were then created, and the mean map was calculated representing the weighted mean regional differences in GM and WM volumes. The default kernel size and statistical thresholds (full width at half maximum [FWHM] = 20 mm, *p* = 0.005, peak height threshold = 1, extent threshold = 10) were used to balance the false positive and negative. A systematic whole‐brain voxel‐based jackknife sensitivity analysis was conducted to test the replicability of the results.

## 3 RESULTS

### 3.1 Included studies

After initially searching, the titles and abstracts with the search strategy, eight VBM studies (Brenneis et al., 2003; D'Agata, Caroppo, Boghi et al., 2011; Goel et al., 2011; Hernandez‐Castillo et al., 2015; Jacobi et al., 2013; Mercadillo et al., 2014; Nave, Ginestroni, Tessa, Cosottini et al., 2008; Nave, Ginestroni, Tessa, Salvatore et al., 2008) were identified as potentially meeting the inclusion criteria. A detailed review of the full text was then performed, and two studies (Hernandez‐Castillo et al., 2015; Jacobi et al., 2013) were excluded due to absence of stereotactic coordinates. As two of the remaining studies used the same group of research subjects, the one with smaller patients sample size (Nave, Ginestroni, Tessa, Salvatore et al., 2008) was excluded. At last, five studies were included. Four from the five studies conducted VBM of both GM and WM volume analyses, while the last one study only studied GM volume difference between SCA2 patients and HCs. A total of 65 SCA2 patients and 124 HCs were included in the GM meta‐analysis. On the other hand, there were 50 SCA2 patients and 109 HCs in the WM meta‐analysis. The technical details of included studies and demographic characteristics of participants are summarized in Table 1. In each of included study, no significant differences were found between two groups in terms of sex and age.

**Table 1:** Characteristics of VBM studies of SCA2 included in the current meta‐analysis

Study	Sample	Sex (M/F)	Mean age (years)	Duration (years)	CAG Repeats	Software	Threshold
Brenneis et al.	SCA2 9	2/7	NA	7.7 ± 2.9	41 ± 3	SPM99	*p* < 0.05 corrected
	HC 27	6/21	NA				
Nave et al.	SCA2 20	10/10	49 ± 12	9 ± 7	39 ± 3	SPM2	*p* < 0.05 corrected
	HC 20	10/10	47 ± 12				
D’ Agata et al.	SCA2 13	10/2	48 ± 10	11 ± 5	39 ± 2	SPM8	*p* < 0.05 corrected
	HC 31	12/19	45 ± 11				
Goel et al.	SCA2 9	7/2	32.7 ± 11.2	7.9 ± 5.4	NA	SPM2	*p* < 0.05 corrected
	HC 31	NA	NA				
Mercadillo et al.	SCA2 15	6/9	37.2 ± 15.9	11.±11.5	44.2 ± 4.4	FSL	*p* < 0.05 corrected
	HC 15	8/7	41.7 ± 13.3				

Note

M/F: male/female; SCA2: spinocerebellar ataxia type 2; VBM: voxel‐based morphometry; mean ± standard deviation.

### 3.2 Regional difference in GM and WM volume

**Figure 1:**
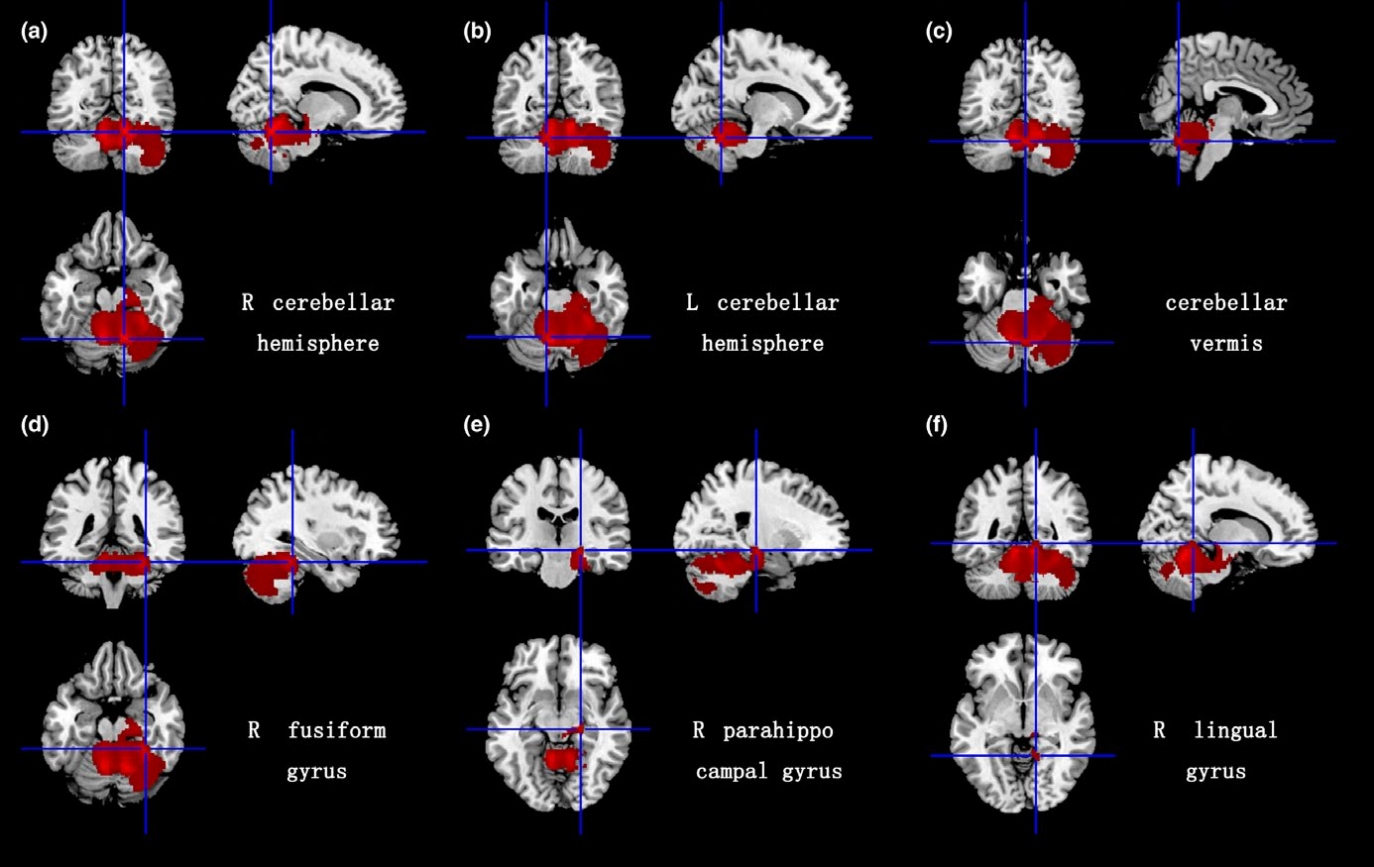
Gray matter atrophy in bilateral cerebellar hemispheres, cerebellar vermis, the right fusiform gyrus, the right parahippocampal gyrus, and the right lingual gyrus. MNI: Montreal Neurological Institute; R: right; L: left

**Figure 2:**
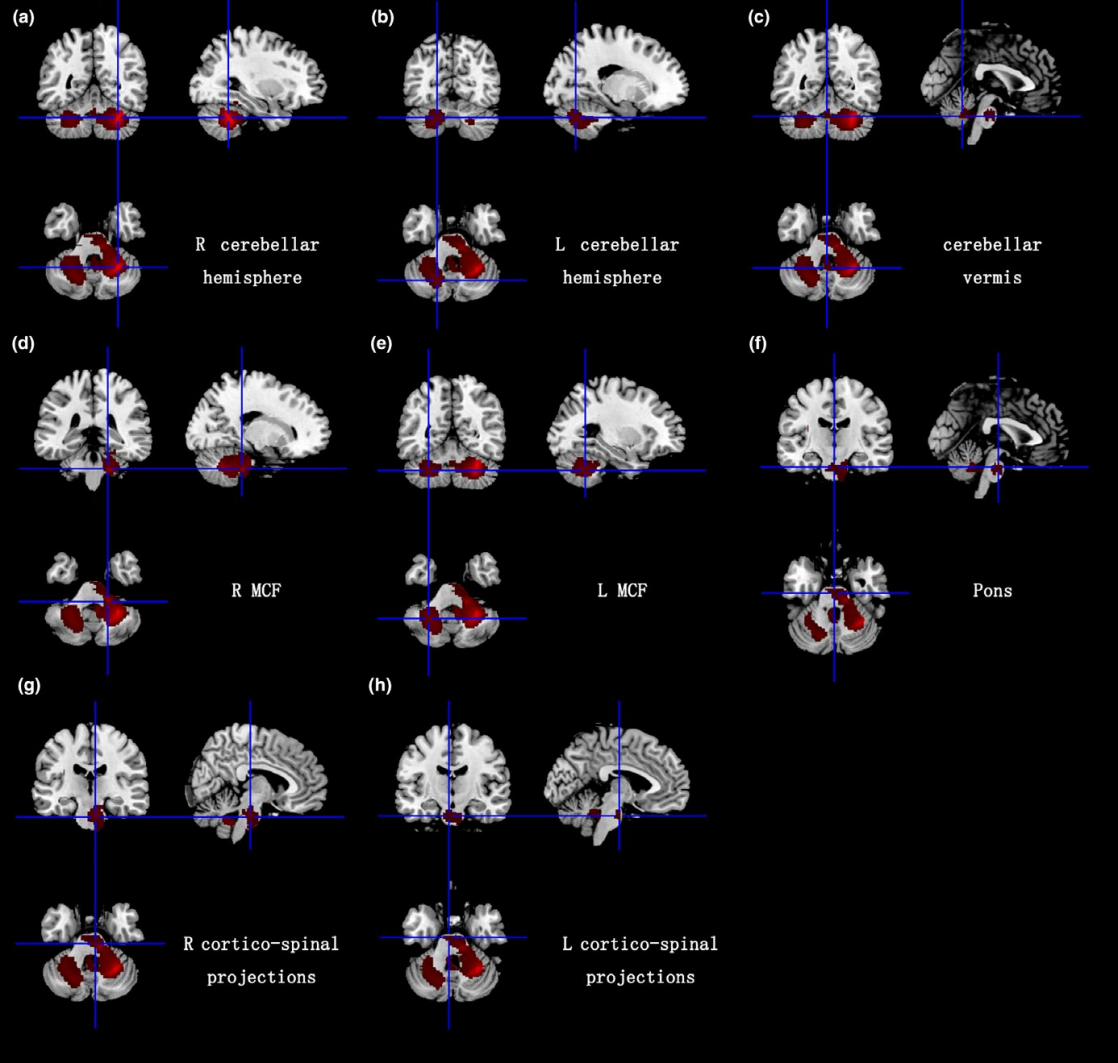
White matter atrophy in bilateral cerebellar hemispheres, cerebellar vermis, middle cerebellar peduncles, pons, and bilateral cortico‐spinal projections. R: right; L: left; MCF: middle cerebellar peduncles

As illustrated in the Figures 1 and 2, significant GM volume reductions were detected in the right cerebellar hemisphere lobule III‐VIII and Crus I/II, the left cerebellar hemisphere lobule IV‐V, the cerebellar vermis lobule IV‐IX, the right fusiform gyrus, the right parahippocampal gyrus, and the right lingual gyrus. The WM volume reductions were observed in the right cerebellar hemisphere lobule III‐VIII and Crus I/II, the left cerebellar hemisphere lobule VI and Crus I/II, the cerebellar vermis lobule III, the bilateral middle cerebellar peduncles (MCP), the pons, the right fusiform gyrus, and the bilateral cortico‐spinal projections. No GM and WM volume increases were found in SCA2 patients compared to HCs. The results from the voxel‐based meta‐analysis are summarized in detail in Table 2.

**Table 2:** Gray matter and white matter volume reductions in SCA2 patients relative to healthy controls

Regions	No. of voxels	Maximum MNI coordinates (x, y, z)	SDM‐*Z* value	*p* Value	Clusters’ breakdown (no. of voxels >50)	Jackknife sensitivity
GM: Cerebellar hemisphere/cerebellar vermis/R fusiform gyrus	9745	12, −60, −20	−3.902	0.000	R cerebellar hemispheric lobule III‐VIII, Crus I/II	5 out of 5
					R fusiform gyrus	5 out of 5
					Cerebellar vermis (lobule IV‐IX)	5 out of 5
					L cerebellar hemispheric lobule IV/V	5 out of 5
					R parahippocampal gyrus	5 out of 5
					R lingual gyrus	4 out of 5
WM: Middle cerebellar peduncles/Cortico‐spinal projections/R Cerebellar hemisphere/pons	3498	30, −52, −34	−6.547	0.000	Middle cerebellar peduncles	4 out of 4
					R cortico‐spinal projections	4 out of 4
					R cerebellar hemispheric lobule III‐VIII, Crus I/II	4 out of 4
					Cerebellar vermis (lobule III)	3 out of 4
					L cortico‐spinal projections	4 out of 4
					Pons	4 out of 4
Middle cerebellar peduncles/L cerebellar hemispheric	1632	−28, −54, −38	−2.624	0.000	Middle cerebellar peduncles	3 out of 4
					L cerebellar hemispheric lobule VI, Crus I/II	4 out of 4

Note

GM: gray matter; L: left; No: number; R: right; SCA2: spinocerebellar ataxia type 2; SDM: seed‐based d mapping; WM: white matter.

### 3.3 Sensitivity analysis

As illustrated in Table 2, the whole‐brain Jackknife sensitivity analysis revealed that GM volume reductions in all areas breaking down from the significant cluster were replicable in five studies, while WM volume reductions in most of all regions were replicable in total of four studies.

## 4 DISCUSSION

As far as we know, the present study is the first quantitative meta‐analysis of VBM studies on both GM and WM volume differences between SCA2 and HCs. Our results identified consistent regions of GM and WM atrophy in SCA2 patients relative to HCs. The consistent GM volume reductions mainly concentrate on cerebellum and a few supratentorial areas (the right parahippocampal gyrus, fusiform gyrus, and lingual gyrus). The reductions in WM volume are detected in cerebellar medulla, cerebellar afferent fibers (middle cerebellar peduncles), and pyramidal system. The high replicability after jackknife sensitivity analysis confirmed the robustness of the findings.

### 4.1 Volume reductions in cerebellum

Atrophy of cerebellum was almost reported in all SCA2 imaging studies and corresponded well to the pathological changes and clinical features, but these studies reported various and inconsistent subregions of atrophy. In the present study, consistent reduced volumes of GM and WM are identified in cerebellar hemispheric lobule III‐VIII and Crus I/II. A previous activation likelihood estimate meta‐analysis confirmed that lobule IV/V and VIII of cerebellum dominate sensorimotor control other than higher‐level functions such as language, spatial, and executive functions (Stoodley & Schmahmann, 2009). Therefore, the atrophy of these regions may result in basic cerebellar signs like ataxia in SCA2 patients. The lobule VI/VII and Crus I of cerebellum are associated with language, verbal working memory, spatial processing, executive functions, and emotional control (D'Agata, Caroppo, Baudino et al., 2011; de Castilhos et al., 2014; Stoodley & Schmahmann, 2009). The atrophy in cerebellar lobule Crus II of cerebellum also plays a role in cognitive impairments, which has been reported (Stoodley & Schmahmann, 2010). A latest study assessed the relationship between cerebellar GM atrophy and neuropsychological scores of the SCA2 patients and demonstrated GM loss in posterior lobules including lobule VI, VIIB, Crus I/II correlated with visuospatial, verbal memory, and executive tasks (Olivito et al., 2018). All these findings above indicated that the atrophy of lobule VI/VII and Crus I/II detected in the current meta‐analysis accounted for cognitive impairments in SCA2. However, only one study did report the cognition impairment of SCA2 patients (Mercadillo et al., 2014).

Reduced volumes in cerebellar vermis (IV‐IX) identified in the current study contains cerebellar vermal lobules VI/VII which have been reported to participate in control of eye movements (Jenkinson & Miall, 2010). This finding is in agreement with ocular movement disorders in SCA2 patients. Atrophy of vermis is also a possibility linked to depression and other psychiatric symptoms in SCA2 patients, as the vermal lobule VII was reliably related to emotion processing (Stoodley & Schmahmann, 2009; Stoodley, Valera, & Schmahmann, 2012).

It should be pointed out that we found larger reduced volumes in the right cerebellar hemisphere than those in the left. Such asymmetry was difficult to explain as most of included studies reported symmetric atrophy between the two cerebellar hemispheres. Perhaps, the coordinates of the regional volume reductions in the left cerebellar hemisphere showed the between‐study heterogeneity, which would influence the result of the SDM approach. Future studies with larger sample sizes may help to verify the asymmetry.

### 4.2 Volume reductions in supratentorial areas

A meaningful result of our study is the GM atrophy in the parahippocampal gyrus, reported once in VBM study of SCA2 (Mercadillo et al., 2014). A previous voxel‐based FDG‐PET reported a metabolic decrease in the parahippocampal area in SCA2 patients compared with SCA3 and SCA6 patients (Wang, Liu, Yang, & Soong, 2007). Another study on SCA2 patients using diffusion tensor imaging (DTI) showed higher mean diffusivity (MD) in WM of the parahippocampal area (Hernandez‐Castillo et al., 2016). In addition, a number of studies on other SCA types like SCA7 and SCA17 have found GM volume reductions in the region of parahippocampal area (Alcauter, Barrios, Diaz, & Fernandez‐Ruiz, 2011; Hernandez‐Castillo et al., 2013; Reetz et al., 2010). It is reasonable to consider the atrophy of parahippocampal gyrus involving in memory formation and spatial analysis in SCA2 patients (Axmacher, Schmitz, Weinreich, Elger, & Fell, 2008; Qin et al., 2009). However, only one included VBM study (Mercadillo et al., 2014) reported the atrophy of parahippocampal gyrus in SCA2 patients, which showed significantly lower scores in cognitive measures than HCs.

The GM atrophy of fusiform gyrus is one of the other important findings in the current study. Fusiform gyrus and the parahippocampal gyrus were reported participating in ventral visual pathway (Op, Beeck, Haushofer, & Kanwisher, 2008) and playing important roles in visual processing and emotion regulation (McLachlan, Bousfield, Howard, & Reeves, 2017; Ward et al., 2014). Some studies have been reported that SCA2 patients developed visual processing and emotion regulation impairment (Fernandez et al., 2000; Pira et al., 2007). A FDG‐PET study revealed reduced metabolism alterations in fusiform gyrus of patients with ataxia‐telangiectasia (Volkow et al., 2014), another ataxia with severe cerebellar vermis atrophy (Verhagen et al., 2012). It may highly indicate the association between fusiform gyrus and cerebellar vermis. The atrophy of fusiform gyrus may be due to the cerebellar vermian deficits as functional connectivity between them has been reported (Sang et al., 2012).

The lingual gyrus has been reported to be involved in the visual recognition and episodic memory (Kukolja, Goreci, Onur, Riedl, & Fink, 2016; Tao et al., 2013; Ward et al., 2014). Significant GM volume reduction in the lingual gyrus was reported in patients with major depressive disorder (Yang et al., 2015). Previous resting state functional MRI studies also observed the associations between the lingual gyrus and psychiatric symptoms such as depression, loneliness, and anxiety (Lan et al., 2015; Liu et al., 2015, 2017). Although psychiatric symptoms were not reported in the five included studies, they are common in SCA2 patients (Alves‐Cruzeiro, Mendonca, Pereira, Almeida, & Nobrega, 2016; Fancellu et al., 2013; Schmitz‐Hübsch et al., 2011). Therefore, the lingual gyrus may also play a role in emotional processing. It also suggests that future studies need more comprehensive assessments for cognition and neuropsychiatric symptoms on SCA2 patients.

In an interesting manner, all these volume reductions of supratentorial areas are located in the right side may implicate the interaction between these supratentorial areas and cerebellum through the ipsilateral networks as predominate right side of cerebellum atrophy was identified in our study and most of these supratentorial regions have been reported to be functional connectivity with different cerebellar subregions (Stoodley & Schmahmann, 2009).

It should be noted that our results did not show significant atrophy of WM in these supratentorial areas, which does not indicate the WM of these region remains normal in SCA2 patients as mild‐to‐moderate damage of WM may not manifest volume reductions. In reality, higher MD in WM of the parahippocampal area was reported (Hernandez‐Castillo et al., 2016). Future researches on WM abnormalities in SCA2 should concern multimode methods including VBM and DTI.

### 4.3 Volume reductions in middle cerebellar peduncles and pons

The middle cerebellar peduncles are the main afferent pathway to the cerebellum, composed by WM fibers originated from the contralateral pontine nuclei (Morales & Tomsick, 2015), which are involved in corticoponto‐cerebellar pathway contributing to motor control (Ramnani, 2006). The previous studies using DTI found more WM microstructural damage in cerebellar tracts including superior cerebellar peduncles (SCP) (Hernandez‐Castillo et al., 2016; Olivito et al., 2017), which are the main efferent pathway from the cerebellum. However, only one included study (D'Agata, Caroppo, Boghi et al., 2011) report atrophy of SCP and it remained normal in the result of current meta‐analysis. It might indicate that the pathological mechanism of the SCP damage was different with which of the MCP damage. The dorsal pons, where pontine nuclei are located, showed volume reductions in the current study, indicating the damage of these pathway in SCA2 patients.

### 4.4 Limitations

Several limitations should be acknowledged in our study. First of all, studies from which we could not extract data and in languages other than English were not included. The heterogeneous and relatively small sample size of included studies might not reflect a standard SCA2 population. Second, the heterogeneity of different methods in VBM studies, including preprocessing protocols, smoothing kernels, and statistical thresholding methods, cannot be ruled out entirely. Third, due to the incomplete information and heterogeneity of included studies, we did not make meta‐regression analysis to explore potential correlations between the GM and WM regional volumes and clinical features such as age, CAG repeats, motor impairments, or cognitive functions. More studies on SCA2 with detailed clinical data in the future will improve the reliability of the meta‐analysis. At last, all of the included studies were cross‐sectional studies that only reflected static alterations of GM and WM volume in SCA2 patients. In reality, a previous longitudinal study using tensor‐based morphometry reported that from baseline to follow‐up, SCA2 patients showed a progressive atrophy of GM and WM in the midbrain and cerebellum, but no volume loss in the supratentorial compartment compared to HCs (Mascalchi et al., 2014). More longitudinal studies with large sample size are required to further understand the neuropathological progression of SCA2.

## 5 CONCLUSIONS

The consistent findings from the present meta‐analysis showed that GM volume reductions in SCA2 patients were mainly focused on cerebellum and a part of cortex in supratentorial regions related to several networks regulating cognitive functions and emotions. The significant WM volume reductions widely existed in cerebellum along with its afferent pathways and pyramidal system. These findings provide morphological basis for further studies in SCA2.
